# Migrating hook wire that travels to the heart via the bloodstream: A case report

**DOI:** 10.1097/MD.0000000000033349

**Published:** 2023-03-31

**Authors:** Kuan-Hua Chen, Chen-Hao Wu, Hao-Ji Wei, Cheng-Wei Chan, Jiun-Yi Hsia

**Affiliations:** a Department of Surgery, Taichung Veterans General Hospital, Taichung, Taiwan; b Department of Radiology, Taichung Veterans General Hospital, Taichung, Taiwan; c Division of Cardiovascular Surgery, Cardiovascular Center, Taichung Veterans General Hospital, Taichung, Taiwan; d Division of Thoracic Surgery, Department of Surgery, Chung Shan Medical University Hospital, Taichung, Taiwan; e School of Medicine, Chung Shan Medical University, Taichung, Taiwan.

**Keywords:** cardiovascular surgery, hook wire migration, intracardiac foreign body, localization hook wire, pulmonary nodules

## Abstract

This report documents the intracardiac migration of a hook wire in a 47-year-old male patient after computed tomography (CT)-guided percutaneous hook wire localization of pulmonary ground-glass opacities.

**Patient concerns:**

The patient underwent CT-guided hook wire localization before video-assisted thoracoscopic surgery (VATS) wedge resection for a pulmonary nodule in the right upper lung field. However, the hook wire was not found in the specimen obtained from the wedge resection. A right upper lobectomy was performed to locate the hook wire; however, it was not found.

**Diagnosis:**

A transesophageal echocardiogram was performed, and the hook wire was found in the left ventricle (LV).

**Interventions:**

The patient subsequently underwent exploratory cardiotomy to remove the foreign body. The patient was admitted to the intensive care unit for postoperative care.

**Outcomes:**

Postoperatively, no complications were observed, and the patient was discharged from the hospital 7 days postoperatively. He received standard lung cancer treatment afterwards.

**Lessons:**

The present case was unique because the hook wire migrated through the bloodstream from the pulmonary vein to the left atrium, before finally reaching the LV. Based on the patient preoperative CT images, the ground glass opacities were proximal to a 2.5 mm wide vein, which drained into the pulmonary vein. The proximity of the hook wire to a blood vessel was reportedly attributed to an increased risk of hook wire migration through the bloodstream. Hematogenous hook wire migration into the heart can result in fatal complications. Early diagnosis and timely removal of the hook wire are recommended to prevent the worsening of this complication.

## 1. Introduction

Computed tomography (CT)-guided percutaneous hook wire localization of pulmonary ground grass opacities before video-assisted thoracoscopic surgery (VATS) is commonly performed. However, hook wire displacement or migration was reportedly the main cause of localization failure or procedural complications.^[1]^ This report documents the intracardiac migration of a hook wire in a 47-year-old male patient after CT-guided percutaneous hook wire localization of pulmonary ground-glass opacities.

## 2. Case report

**Figure 1.**
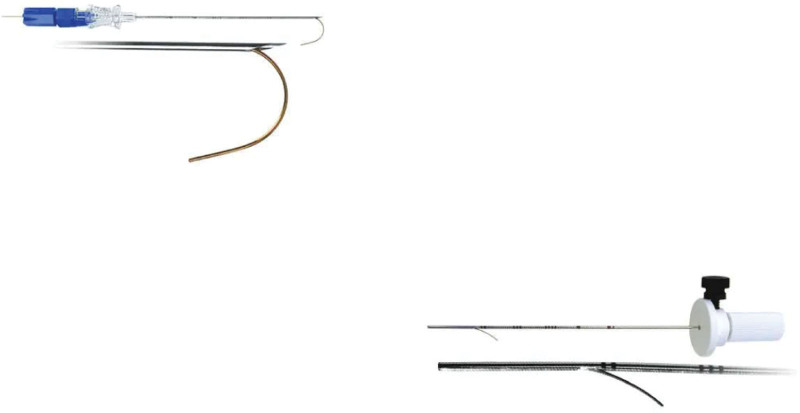
Argon Breast Localization Needle (Argon Medical, TX) used for the localization of pulmonary nodular lesions before video-assisted thoracoscopic surgery.

A 47-year-old male patient underwent CT-guided hook wire localization (Argon Breast Localization Needle (Argon Medical, TX), Fig. 1) before VATS wedge resection for a pulmonary nodule at the right upper lung field. Hook wire localization was performed in the CT room. Then, the patient was sent to the operation room for surgery. However, the hook wire was not found in the specimen obtained from the wedge resection. A right upper lobectomy was performed to look for the hook wire, although it was not found. Therefore, a transesophageal echocardiogram was performed, and the hook wire was found in the left ventricle (LV). Upon consulting a cardiologist, emergent transcatheter removal of the wire was performed. The tip was successfully snared, although the hook wire was not removed. The patient was admitted to the intensive care unit for postoperative care, and he remained stable.

**Figure 2.**
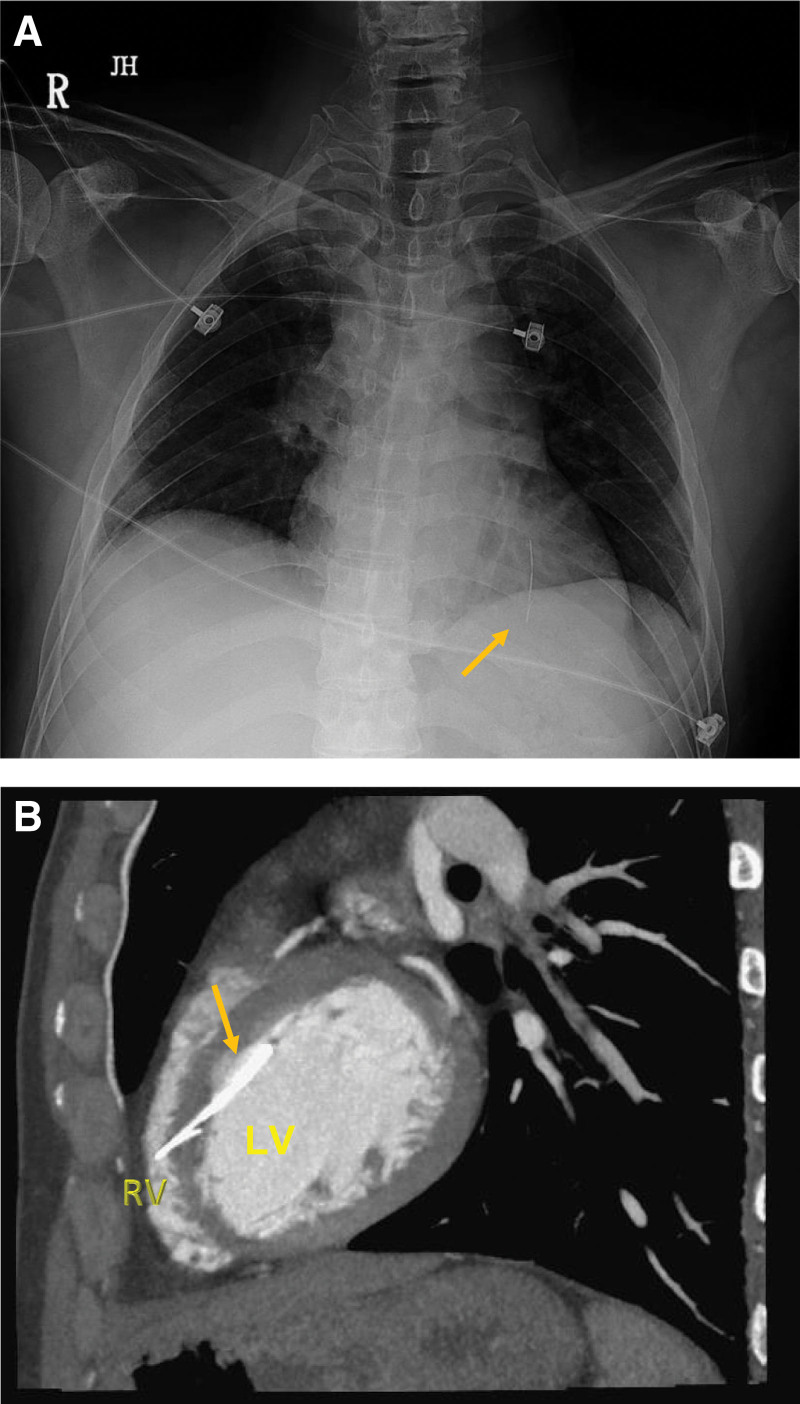
(A) A chest radiograph on the day of admission reveals a linear radiopaque density superimposed in the lower mediastinum. This is suggestive of a foreign body in the left ventricle. (B) Preoperative multi-detector computed tomography reveals a metallic foreign body in the left ventricle that penetrated the interventricular septum.

Several days later, a chest X-ray showed a linear foreign body in the mediastinum (Fig. 2A). Multi-detector CT revealed a metallic foreign body in the LV, which was compatible with the previous finding of a hook wire. The hook wire penetrated the interventricular septum (Fig. 2B), and the tip of the wire was located in the right ventricle (RV). Echocardiography showed normal systolic wall motion with an ejection fraction of 53.3%. A hyperechoic foreign body across the intraventricular septum penetrated from the LV to the RV. No pericardial effusion was observed.

**Figure 3.**
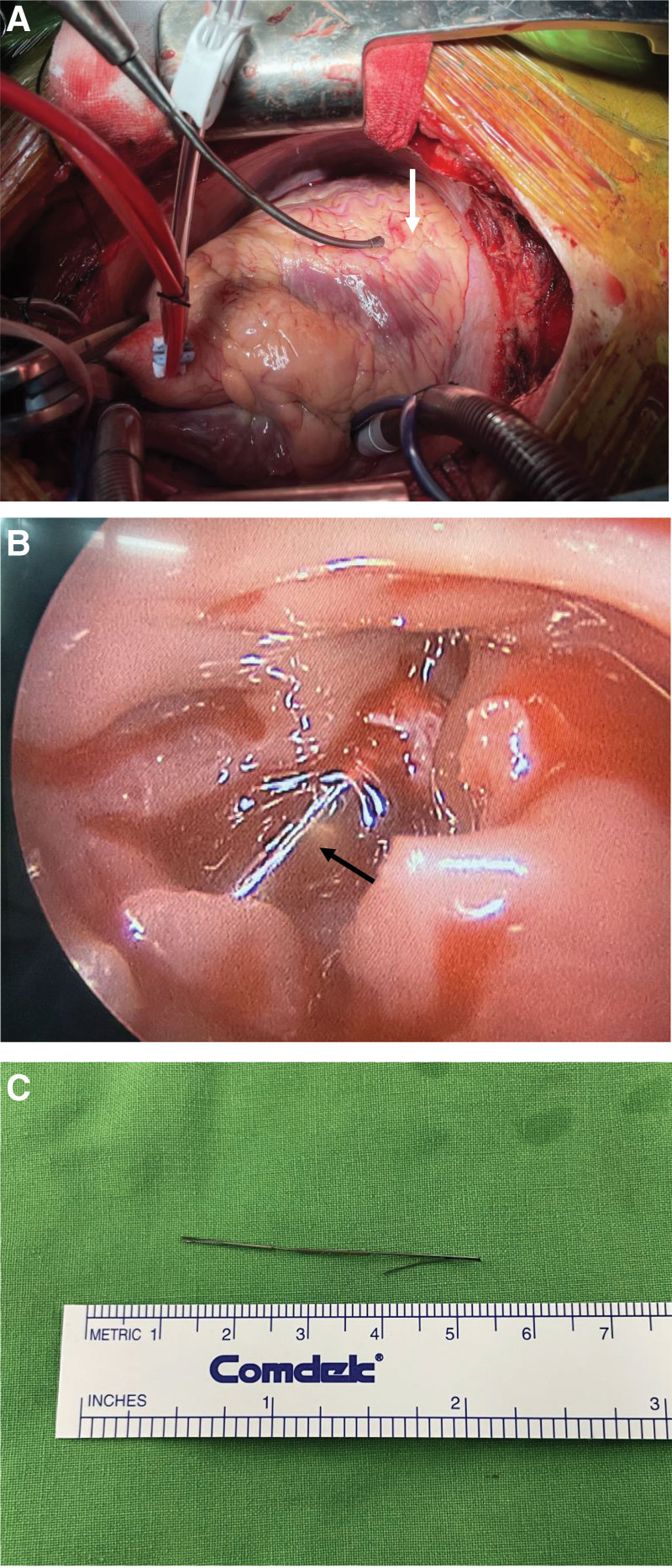
(A) Upon opening the pericardial sac, mild ecchymosis (epicardial abrasion) over the anterior wall of the RV is observed. (B) A right atriotomy was performed to gain access to the heart. After transecting a small trabeculation, the tip of the wire can be observed in the right ventricle by the scope, penetrating the inter-ventricle septal wall. (C) The 4 cm wire was removed from the right ventricle. RV = right ventricle.

Exploratory cardiotomy for foreign body removal was performed on postoperation day 12. The intraoperative transesophageal echocardiography was used to assess wall motion and the valvular structures. Upon opening the pericardial sac, mild ecchymosis (epicardial abrasion) was observed over the anterior wall of the RV (Fig. 3A). The pericardial fluid was clear. A cardiopulmonary bypass was established via standard ascending aorta and bicaval cannulation. The heart was arrested with a single dose of cardioplegia, and a right atriotomy was performed. After transecting a small trabecula, the tip of the wire, penetrating the intraventricular septum, was appreciated distally in the RV (Fig. 3B). With direct visualization, the wire was removed successfully (Fig. 3C). postoperative transesophageal echocardiography was performed, and it showed an adequate LV ejection fraction of 68% to 72%, minimal mitral regurgitation, no residual foreign body, and no abnormal shunt at the interventricular septum.

Postoperatively, the patient was immediately extubated. He stayed in the surgical intensive care unit for 2 days, and no complications were observed. The chest drain was removed shortly after the operation. He was discharged from the hospital 7 days postoperatively. The follow-up examination yielded unremarkable results. Pathologic examination of the specimen, obtained from the right upper lobectomy, revealed minimally invasive adenocarcinoma, nonmucinous type, well-differentiated. The patient received the standard lung cancer treatment.

## 3. Discussion

Thoracoscopic surgery has become a standard diagnostic and treatment modality for pulmonary lesions because it decreases postoperative morbidity and hospital stay. Various preoperative techniques for localization and complete excision have been developed. CT-guided preoperative hook wire localization is a convenient and reliable method.^[2]^

The most common complications of this procedure include pneumothorax, pulmonary hemorrhage, hemoptysis, and postoperative pain. There have also been reports on wire fragmentation and migration to other sites.^[3,4]^ Ichinose et al reported the dislodgement of hook wires in 2 patients among 178 cases involving ground-glass opacities and 322 involving solid lesions, treated via CT-guided hook wire localization (0.4%).^[2]^

In particular, the dislodgement of the hook wire into the intrapleural, intrapericardic, intramyocardial, and intravascular compartments results in life-threatening disability, requiring prompt management and removal. The hook wire rarely migrates to the heart.^[5,6]^ The present case was unique because the hook wire migrated through the bloodstream from the pulmonary vein to the left atrium, and it ended up in the LV. The hook wire penetrated the septum and entered the RV. In cases, wherein the wire tip cannot be visualized in the RV, removal via the right atrium approach is the most feasible option. The wire needs to be pushed from the LV (transmitral valve) into the RV. This facilitates the removal via the biatrial approach.

The abrasion stain on the RV epicardium (Fig. 3A) indicated the possibility of penetration of the free wall, followed by cardiac tamponade. Moreover, mural thrombi formation and endocarditis may occur as a result of the prolonged presence of a foreign body in the heart.^[7]^

**Figure 4.**
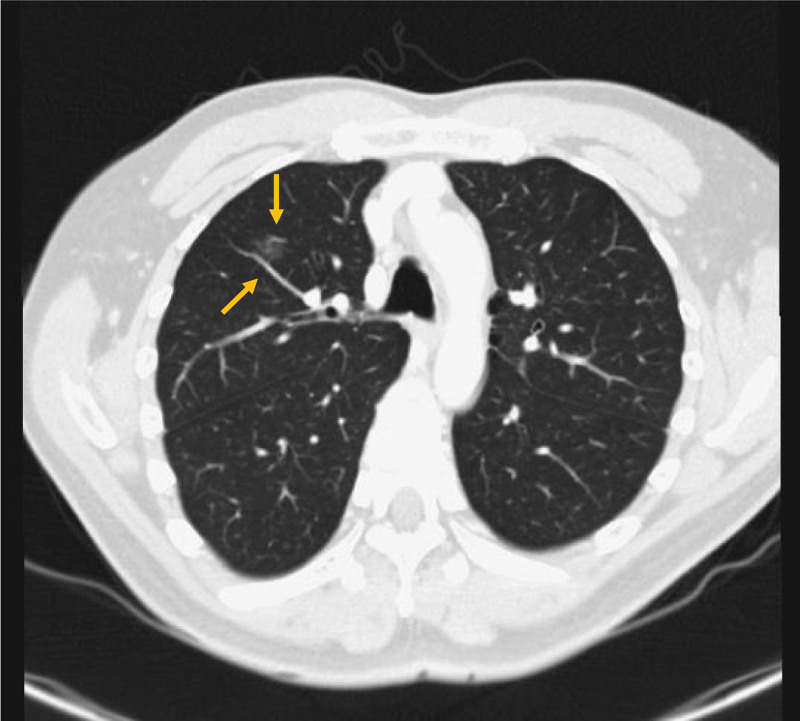
The patient previous chest computed tomography reveals that the ground grass opacity is located proximal to a vein that drains back to the pulmonary vein; the diameter of the vein is 0.25 cm.

Based on the patient preoperative CT images, the ground glass opacities were proximal to a vein, draining into the pulmonary vein. Its proximity to a blood vessel was reportedly attributed to an increased risk of hook wire migration through the bloodstream. The diameter of the vein was 2.5 mm, and a straight line segment was observed (Fig. 4). These characteristics (proximity to a straight vessel with a diameter >2.5 mm) increase the risk of wire migration. Performing hook wire localization in the hybrid operation room should be considered to reduce the interval between localization and VATS, thereby decreasing the risk of migration.^[8]^ Applying percutaneous lipiodol injection or micro coil is another alternative method to localize the pulmonary nodule.^[9,10]^ It has a higher success rate and lower incidence of complications, including dislocation, migration, and penetration into nearby soft tissues.^[10]^

Hematogenous hook wire migration into the heart results in fatal complications. The beating of the heart causes the penetration of the hook into the myocardium and other tissues, resulting in cardiac tamponade, hemothorax, and pneumothorax. Early diagnosis and timely removal of the hook wire were recommended to prevent the worsening of this complication. In cases involving a lesion, proximal to a vessel with a diameter >2.5 mm, alternative localization methods should be considered.

## Acknowledgments

We wish to thank various people for their contribution to this case report; Dr Chen-Hao Wu, for his valuable technical support on this case report; Ms Hsin-Yi Huang, staff member of the Cardiovascular Surgery Office, for her help in handling documents and project applications. Special thanks should be given to Dr Hao-Ji Wei, my case study supervisor for his professional guidance and valuable support, and to Dr Cheng-Wei Chan and Dr Yi-Wen Chen for their useful and constructive recommendations on this case report. Editorial support, in the form of medical writing, assembling and creating high-resolution images based on authors’ detailed directions, collating author comments, copyediting, fact checking, and referencing, was provided by the Editage company.

## Author contributions

**Conceptualization:** Hao-Ji Wei, Jiun-Yi Hsia.

**Data curation:** Kuan-Hua Chen, Hao-Ji Wei, Jiun-Yi Hsia.

**Investigation:** Chen-Hao Wu.

**Writing – original draft:** Kuan-Hua Chen.

**Writing – review & editing:** Hao-Ji Wei, Cheng-Wei Chan.
